# Risk Factors and Circulating Subtypes of *Cryptosporidium* spp. and *Giardia duodenalis* in Hospitalized Children in Mozambique

**DOI:** 10.3390/microorganisms13010196

**Published:** 2025-01-17

**Authors:** Ofélia Nhambirre, Maria Luísa Lobo, Idalécia Cossa-Moiane, Adilson Bauhofer, Nilsa de Deus, Olga Matos

**Affiliations:** 1 Unidade de Parasitologia Médica, Grupo de Parasitas Oportunistas/VIH e Outros Parasitas, Global Health and Tropical Medicine, GHTM, Associate Laboratory in Translation and Innovation Towards Global Health, LA-REAL, Instituto de Higiene e Medicina Tropical, IHMT, Universidade NOVA de Lisboa, 1349-008 Lisboa, Portugal; yufenha@yahoo.com.br (O.N.); omatos@ihmt.unl.pt (O.M.); 2 Instituto Nacional de Saúde (INS), EN1, Bairro da Vila-Parcela n°3943, Distrito de Marracuene, Maputo 264, Mozambique; idaleciacossa@yahoo.com.br (I.C.-M.); adilsonbauhofer@gmail.com (A.B.); ndeus1@yahoo.com (N.d.D.); 3 Instituto de Saúde Ambiental, Faculdade de Medicina, Universidade de Lisboa, 1649-028 Lisboa, Portugal; 4 Laboratório Associado TERRA, 1349-017 Lisboa, Portugal; 5 Institute of Tropical Medicine, 2000 Antwerp, Belgium; 6 Departamento de Ciências Biológicas, Universidade Eduardo Mondlane, Maputo 3453, Mozambique

**Keywords:** *Cryptosporidium* spp., *Giardia duodenalis*, diarrhea, children, Mozambique

## Abstract

*Cryptosporidium* spp. and *Giardia duodenalis* are important diarrheal agents in children in developing countries. Little is known about their molecular epidemiology; as such, the objective of this study was to determine the risk factors and genetic diversity of both parasites in diarrheal samples in Mozambique. In this study, two nested PCRs targeting *ssurRNA* and *gp60* genes were used for genetic diversity of *Cryptosporidium* spp. and *b-giardin* for *G. duodenalis*. Sociodemographic and clinical characteristics were obtained through questionnaires. The location (odds ratio [OR] 3.499), mother’s education level (OR 2.150) and age were significant factors for acquiring infection by *Cryptosporidium* spp. (*p* < 0.05). *Cryptosporidium hominis* was the predominant (77.8%) species. Four families (three *C. hominis* and one *C. parvum*) were identified, with the highest for Ib (73.9%), followed by Id (13%), Ia (8.7%) and IIc (4.3%). The location (*p*-value < 0.001), drinking untreated water (*p*-value = 0.04) and living in masonry houses (*p*-value = 0.002) were identified as risks associated with *G. duodenalis* infection. Assemblage A was the dominant type (65.2%). Among the subassemblages of assemblage A, AII was the most frequent (86.6%), followed by AIII (6.6%). For assemblage B, subassemblages BIII (87.5%) and BIV (12.5%) were identified. The dominance of the subtype IbA9G3 of *C. hominis*, as well as the AII subassemblage of *G. duodenalis*, seems to indicate that the transmission of both protozoa occurs mainly through the anthroponotic route.

## 1. Introduction

Diarrheal disease remains a leading cause of illness and death among children under five in Sub-Saharan Africa, with severe short- and long-term impacts [1]. Protozoa such as *Cryptosporidium* spp. and *Giardia duodenalis* are major contributors to these infections [2,3]. According to the Global Enteric Multicenter Study (GEMS), *Cryptosporidium* is the second most common pathogen in this age group, responsible for 14.7% of diarrheal cases, following rotavirus at 27.8% [4]. Approximately 7.6 million diarrhea cases annually are attributed to *Cryptosporidium,* with 2.9 million occurring in Sub-Saharan Africa [5]. Estimates suggest that approximately 7.6 million cases of diarrhea annually are attributed to *Cryptosporidium*, with 2.9 million of these occurring in Sub-Saharan Africa alone [5].

*Cryptosporidium* spp. has a variety of animal and human hosts. Young children and immunocompromised individuals are particularly vulnerable due to their higher exposure to contaminated environments and weaker immune defenses. Infections often result in diarrhea, undernutrition, stunted growth, and cognitive impairments, significantly affecting child health [2,4]. The high rate of asymptomatic carriers also highlights the urgent need for research and targeted interventions to address this public health challenge in low-resource settings [6].

*Giardia* is a flagellated zoonotic protozoan responsible for up to 280 million infections annually [7]. Its pathogenicity is not fully understood [8], but symptomatic infections (giardiasis) can cause acute or chronic diarrhea. Severity and duration are influenced by factors such as host immune response, parasite strain, and infectious dose. While giardiasis is typically self-limiting in immune-competent individuals, it can lead to anorexia, impaired growth, and poor nutritional status in children under five [9,10]. *Giardia* has been associated with various wild animals (rats, beavers, chimpanzees, gorillas, buffaloes and impalas) [11,12] and domestic animals (cattle, pigs, dogs, cats and birds) [13].

The epidemiology of *Giardia* and *Cryptosporidium* is intricate due to their zoonotic potential and the various transmission routes involved [14]. Understanding these complexities is crucial for developing effective prevention strategies. Consequently, prophylaxis must be based on three key pillars: environmental, epidemiological, and etiological control, all framed within a One Health approach. This integrative framework enables more targeted risk management, which is essential for mitigating the public health impacts of these protozoa.

The first case of infection by *Cryptosporidium* spp. in Mozambique was reported in 1999 by Clavero in diarrheal stools from an adult patient with human immunodeficiency virus (HIV)/acquired immunodeficiency syndrome (AIDS) (Chókwè, Gaza province, Mozambique) [15]. From this period onwards, it became a focus of research, but there is still a lack of molecular studies. Molecular studies have been widely conducted worldwide to assess the genetic diversity of *Cryptosporidium* species and *G. duodenalis* assemblages, as they are morphologically indistinguishable [16,17]. This information is important to assess the distribution and to understand the zoonotic potential of species, genotypes and subtypes as well as the transmission routes [18]. The genetic diversity of *Cryptosporidium* spp. and *G. duodenalis* demand the use of genotyping tools. Several molecular markers allowed the characterization of *Cryptosporidium* spp. [16,18,19], including the genes encoding *ssurRNA* and *gp60* [18]. Based on the *ssurRNA*, about 20 *Cryptosporidium* species and many genotypes of unknown species status have been described as pathogenic to human beings, with emphasis on *Cryptosporidium hominis* and *Cryptosporidium parvum* for being the most common. On the other hand, the *gp60* gene, characterized by a high degree of polymorphism, revealed the presence of several families of subtypes of *C. hominis, C. parvum, Cryptosporidium ubiquitum, Cryptosporidium andersoni* and *Cryptosporidium meleagridis* [18,20].

Regarding *G. duodenalis*, eight distinct genetic variants (assemblages), A–H, were identified, and the genotypes A and B are considered pathogenic for humans [21]. The most common genotyping tool markers are triose-phosphate isomerase—*tpi* gene, beta-giardin *bg* and glutamate dehydrogenase—*gdh*, either alone or using a combination of two or three loci [22]. These three markers also allowed 10 multilocus genotypes (MLG) to be defined (AI-1 and 2, AII-1 to 7 and AIII-1) for genotype A and genotype B, BIII-1 and BIV-1 [23].

Epidemiological research on *Cryptosporidium* and *Giardia* infections in Mozambique has progressed, but significant gaps remain regarding the molecular diversity of these parasites. So far, five genotyping studies on *G. duodenalis* have been conducted in Nampula, Zambézia, Gaza and Maputo provinces [24,25,26,27,28]. In Nampula, a study involving 831 children identified *G. duodenalis* as the most common parasite (23.9%) in stool samples using parasitological and molecular analyses based on the *bg* gene. In Zambézia, 807 asymptomatic and 286 symptomatic children underwent molecular characterization using PCR-based methods targeting the *bg*, *tpi* and *gdh* genes and Sanger sequencing. In Gaza, stool samples from 99 patients positive for HIV and/or tuberculosis were analyzed using microscopy, real-time PCR and *Giardia* genotyping based on the *gdh* gene. In Maputo (Manhiça district), studies on 757 stool samples from children under five revealed assemblage B (90%) as being dominant, with assemblage A (8%) and mixed infections also detected by multiplex PCR. Another study found *G. intestinalis* in 28.5% of 291 stool samples using nested PCR targeting the *tpi* gene. For *Cryptosporidium*, five studies conducted in Mozambique [5,6,26,29,30] involved children and adults with diarrhea. Methods included microscopy, PCR-RFLP, immunoassays and *gp60* sequencing to identify species and subtypes. These methods were consistently applied depending on the study’s objectives.

Studies using PCR detection have identified predominant genotypes, indicating potential zoonotic and human transmission routes. However, genotyping has been limited to specific age groups, leaving gaps in understanding the genetic diversity and transmission dynamics among older children. This study is the first to comprehensively intend to evaluate the genetic variability of *Cryptosporidium* spp. and *G. duodenalis* across Mozambique’s southern, central and northern regions, including Maputo, Sofala, Zambézia and Nampula. It enrolls children up to 14 years old with gastrointestinal symptoms, exploring diverse geographic and demographic contexts. The research aim emphasizes the need for broader investigations to understand the public health impact and local epidemiology of these infections. Its main objectives were to identify risk factors and assess the genetic variability of *Cryptosporidium spp.* and *G. duodenalis* in children with diarrhea.

## 2. Materials and Methods

### 2.1. Study Area, Population and Inclusion Criterion

This study is a part of an ongoing Mozambican project “National Diarrhea Surveillance (ViNaDia)”, conducted in six health units in four provinces of Mozambique: Maputo (*Hospital Central de Maputo—HCM*, *Hospital Geral José Macamo—HGJM* and *Hospital Geral de Mavalane—HGM*), Sofala (*Hospital Central da Beira—HCB*), Zambézia (*Hospital Provincial de Quelimane—HPQ*) and Nampula (*Hospital Central de Nampula—HCN*), corresponding to the three geographic regions of the country (south, center and north). Samples were collected from May 2014 to December 2019.

The inclusion criteria were children aged up to 14 years, with diarrhea (defined as three or more bowel movements in the last 24 h) from ViNaDia [31].

### 2.2. Specimen and Clinical Data Collection

A total of 1424 stool samples were collected from children with diarrhea, presented in outpatient clinics or hospitalized, whose parents or guardians visited the six health units and voluntarily agreed to participate in the study. Fresh stool samples were stored in containers with screw caps, properly identified and kept between 2 °C and 8 °C. These samples were sent on the same day to the central laboratory of the National Institute of Health-Mozambique (INS) from the collection sites located in Maputo city for microscopic analysis. Samples from the center and north regions were stored at −20 °C and sent to the INS once a week. Informed consent was obtained from the child’s parents or primary caregiver, and then a questionnaire was applied to assess potential risks for *Cryptosporidium* spp. (gender, age, type of food, water source, education, marital status, agricultural practice and child caretaker).

### 2.3. Microscopic Detection of Cryptosporidium spp. and G. duodenalis

A total of 1424 fresh samples and the samples previously concentrated by the formol–ether method (OMS) were used to prepare thin smears and then stained with the modified Ziehl–Neelsen staining method [32]. Briefly, two smears were added in the slide, one from the fresh stool and the second from the formol–ether sediment. The smears were fixed with methanol and stained by adding a fuchsin solution, followed by an alcohol–acid solution 3%. Finally, malachite green solution was added, and the preparation was examined under the optical microscope at objective 100×, after adding immersion oil (ROTH, Karlsruhe, Germany) to the slides for observation. Cysts and trophozoites of *G. duodenalis* were screened by Lugol staining to the concentrate resulting from the formalin–ether technique [32]. This technique was performed using a suspension of 1 g of stool in 10% formalin and ether and centrifugated at 2500 rotations per minute. The Lugol iodine solution was added to the resulting pellet and an observation was performed with an optical microscopy in the 10× objective and then switched to 40×. Data obtained by the parasitological diagnostic methods were previously reported by the authors [33].

### 2.4. Genomic DNA Extraction

For genetic diversity analysis, in addition to the *Cryptosporidium* spp. and *G. duodenalis*-positive samples detected by microscopy and previously reported by the authors [33], positive samples previously tested by ELISA (TechLab, Inc, Blacksburg, VA, USA) and reported in Bauhofer et al. [34] were also included. In this way, the genomic DNA of *Cryptosporidium* spp. (69 positive stool samples) and *G. duodenalis* (79 positive samples) was extracted using the commercial QIAgen FastDNA SPIN kit (Quiagen. Hilden, Germany) according to the manufacturer’s instructions [35]. Briefly, 220 mg of stool sample was placed in a microtube, ASL buffer was added and centrifuged and then the supernatant recovered to a new microtube where an inhibitor tablet was added. Proteinase K and the supernatant from the previous step and the AL buffer were added to a new microtube. Ethanol (96–100%) was added to the lysate and centrifuged. The pellet was washed twice with Buffer AW, and finally, Buffer AE was added for DNA elution and afterward stored at −20 °C. After DNA extraction, the amplicons were sent to Portugal for sequencing. Subsequently, genotyping and phylogenetic analyses were performed to characterize the genetic profiles.

### 2.5. Cryptosporidium spp. Identification

The amplicons were subjected to the sequential amplification of the gene *ssurRNA* by nested PCR for species identification. The amplification of the *ssurRNA* gene was performed in a first reaction with the primers CrySSUF1 and CrySSUR2, followed by a second reaction with the pair CrySSUF3/CrySSUR4 [36]. The expected size of the amplicon in the first reaction was 1325 bp and in the second was 831 bp [36]. The two PCR reactions were performed in a final volume of 25 μL, containing 21 μL mixes and 4 μL DNA template. The amplification conditions for both PCR reactions were identical: 1 cycle of initial denaturation at 95 °C, 5 min; 34 cycles at 95 °C, 30 s (denaturation), 55 °C, 45 s (annealing), and 72 °C, 1 min (extension); and the final extension phase at 72 °C, 10 min.

All the oligonucleotides (primers) used in the PCR procedures were purchased and synthesized by StabVida (Lisbon, Portugal). A thermocycler Biometra T1 (Seattle, WA, USA) was used to perform all the PCR analyses in this study.

### 2.6. Cryptosporidium hominis and C. parvum Subtyping

The subtyping of the two main infective species for humans was also performed by nested PCR targeting the *gp60* gene, using primers Crygp60F1 and Crygp60R1 in the primary reaction and gp60F2 and gp60R2 for the secondary reaction [37]. The expected sizes of the products for the first and second reactions corresponded to 460 bp and 360 bp, respectively [37].

PCR amplification was performed in a total volume of 25 μL, containing 21 μL mix and 4 μL DNA template. The amplification conditions were as follows: 1 cycle of initial denaturation at 95 °C for 5 min; 34 cycles of denaturation at 94 °C for 45 s, binding at 50 °C for 45 s, and extension at 72 °C for 1 min; and final extension at 72 °C for 10 min.

Each PCR run included a negative control (deionized water) and positive control (genomic DNA from *Cryptosporidium* spp., oocysts from a fecal specimen kindly provided by the *Instituto de Higiene e Medicina Tropical* (IHMT), laboratory of parasitology, Lisbon, Portugal). Products were visualized in 1.5% agarose gels using 1× SYBR Safe DNA gel Stain (Invitrogen, Carlsbad, CA, USA), and sequencing was used for the identification of species and subtypes.

### 2.7. Molecular Typing of G. duodenalis

For the detection of *G. duodenalis* DNA, the *bg* gene was amplified by nested PCR according to other authors [38].

The amplification reactions were performed in a total volume of 25 μL containing the first reaction 7 μL of water, 2.0 μL of 10× of the buffer (Tris-HCl 100 mM-pH 8.8 and KCl 500 mM), 1.5 μL at 50 mM of MgCl_2_, 2 μL of DNTP from 10 mM, 1 μL of each primer at 10 pmol/μL, 2 μL of BSA at 10 mg/μL, 0.5 U of DNA polymerase (5 U/μL) (Thermo Scientific, Waltham, MA, USA) and 10 μL of the sample DNA. In the second reaction, 10 μL of water, 3 μL of buffer, 1.5 μL of each initiator, 1 μL of BSA and 5 μL of the first reaction product were used, with the other reagents having the same amounts of the first reaction.

The primers GiaFW1 and GiaRv1 were used for the first reaction and the pair Gi-aFW2/GiaRv2 for the second reaction under the following conditions: 1 cycle of initial denaturation at 95 °C, and 34 cycles of the following: denaturation for 5 min, 45 s of Bond 65 °C/55 °C, 1 min extension at 72 °C and 10 min final extension at 72 °C. For the first reaction, fragments of 753 bp were expected, and for the second reaction, fragments of 511 bp were expected. The conditions of amplification in the first and second reactions are the same as the exception of the annealing temperature (first reaction: 65 °C; second reaction: 55 °C).

### 2.8. Sequencing of ssurRNA and gp60 and bg PCR Amplicons

All PCR-positive products were purified using the PureLink™ Quick Gel Extraction and PCR Purification Combo kit (Invitrogen, Carlsbad, CA, USA) according to the manufacturer’s instructions [39]. The purified amplicons were sequenced in both directions using primers CrySSU3/CrySSU4 for the ssurRNA PCR product, gp60F2/gp60R2 for the *gp60* PCR product of *Cryptosporidium* spp. and GiaF2/GiaR2 for the *bg* gene amplicons of *G. duodenalis*. Sequencing services were obtained from StabVida (Lisbon, Portugal), which uses the commercial BigDye^®^ Terminator v3.1 kit. Sequencing chromatograms were generated on an ABI PRISM 3130xl Genetic Analyzer (Applied Biosystems, Carlsbad, CA, USA) under standard conditions for Sanger sequencing.

The chromatograms were analyzed by the authors with the software ChromasPro v1.5 (http://www.technelysium.com.au/ChromasPro.html, (accessed on 8 October 2022) to check for accuracy and quality. Subsequently, the high-quality sequences obtained were aligned manually and compared with reference sequences available in the GenBank database (http://www.ncbi.nlm.nih.gov/genbank/, (accessed on 8 October 2022) using the Basic Local Alignment Search Tool (BLASTN) (http://blast.ncbi.nlm.nih.gov/Blast.cgi, (accessed on 8 October 2022). Multiple alignments of all sequences for each locus were performed using the Multiple Sequence Alignment program by Florence Corpet (MultAlin) (http://multalin.toulouse.inra.fr/multalin/, (accessed on 8 October 2022), with manual adjustments made to eliminate inaccuracies that could interfere with the final interpretation.

Phylogenetic trees of the nucleotide sequences of the genes *gp60* and *bg* gene were performed using the Molecular Evolutionary Genetics Analysis Version 11 (MEGA 11) software package [40]. Phylogenetic relationships were inferred using the Maximum Likelihood method ref. Genetic distances were calculated using the 2-parameter Kimura model ref. Branch reliability was assessed using bootstrap analysis using 1000 replicates. *Cryptosporidium meleagridis and Giardia muris* (accession no. KJ 210619) were used as out-groups.

### 2.9. Statistical Analysis

Data were analyzed using the Statistical Package for the Social Sciences, Armok, NY, USA: IBM Corp, 2011, version 26.0 (IBM SPSS). Initially, bivariate analyses were conducted using chi-square tests and Fisher’s exact test to explore associations between potential risk factors and infections by *Cryptosporidium* spp. and *G. duodenalis*. Odds ratios (OR) and 95% confidence intervals (95% CI) were calculated to describe risk factors. Variables with a *p*-value < 0.20 in the bivariate analyses were further included in multivariate logistic regression models to control for potential confounding factors. The final regression models were built using a stepwise forward selection approach, guided by field knowledge and theoretical relevance of the following variables: gender, age groups, province, type of food, water source, the mother’s education, the child’s caregiver, marital status and type of housing. Statistical significance was set at *p*-value < 0.05 for all analyses.

## 3. Results

### 3.1. Sociodemographic Description of Study Participants

A total of 1424 stool samples were screened by microscopy for parasites, in a previous study reported by the authors [33]. Of these, 58.3% (830/1424) were collected from males, and 47.9% (682/1424) were from individuals below 12 months of age. Most of the children were recruited in Maputo province (52.3%, 745/1424), while the province of Sofala comprised the lowest numbers (5.5%, 78/1424). The Hospital Central de Nampula was the one that had the most cases of diarrhea during the study period, with 31.1% (443/1424) of cases. Data are summarized in Table 1.

### 3.2. Prevalence and Risk Factors for Cryptosporidiosis and Giardiasis

*Cryptosporidium* spp. was identified in the feces of 8.1% (115/1424) of children with diarrhea using microscopy, as previously reported by the authors [33]. Its occurrence was higher in children aged between 12 and 23 months at 9.6% (46/481) (*p*-value = 0.021), from Sofala (10.3%, 8/78) (*p*-value = 0.038) and cared for by mothers without literacy (13.1%, 22/168) (*p*-value = 0.036). Data are summarized in Table 1.

*Giardia duodenalis* was detected in the feces of 1.3% (19/1424) of children with diarrhea, as previously reported by the authors [33]. Children recruited in Maputo (2.4%, 18/745) (*p*-value = 0.001) whose families did not do any water treatment (1.4%, 12/881) (*p*-value = 0.040) and who lived in brick house houses (2.1%, 19/884) had significant infections by *G. duodenalis* (*p*-value = 0.002) (Table 1).

The associated factors for *Cryptosporidium* were then confirmed in the logistic regression analysis. The location was a positive predictor of *Cryptosporidium*. In this view, children from Sofala were at greater risk of becoming infected when compared to children from Nampula (OR 3.499, CI: 1.095–11.185). The mother’s education level was also a significant factor for acquiring *Cryptosporidium* spp. infection, presenting more chance of infection in children of non-literate mothers (OR 2.150, CI: 1.252–3.690) when compared to children of mothers with secondary education (Table 2). From this analysis, it was also found that with each month of age, *Cryptosporidium* infection reduced by 2.8%. A logistic regression model was not applied for *G. duodenalis* infection due to its low frequency in the studied population (1.3%), which could lead to confounding.

### 3.3. Genotyping and Subgenotyping of Cryptosporidium spp.

All the 69 isolates were tested for both *ssurRNA* and *gp60* genes. Genotype data were obtained in 18 isolates for the *ssurRNA* genes. The remaining samples did not give any amplification.

*Cryptosporidium hominis* (77.8%, 14/18) was the most frequent type detected. The remaining 22.2% (4/18) of the sequences were identified as *C. parvum/C. hominis*; hence, no exact species was assigned to each of them; on the other hand, none of them was like another human infective species.

Subgenotyping data, based on the *gp60* gene, were obtained in 23 of the positive PCR samples. The other sequences were excluded due to lack of amplification or inadequate quality. Using BLAST analysis, the sequences revealed the presence of three families of subtypes Ib 73.9% (17/23), Id 13.0% (3/23) and Ia 8.7% (2/23) of *C. hominis* and one family of *C. parvum,* IIc 4.3% (1/23).

The four isolates previously identified as *C. parvum/C. hominis* by the *ssurRNA* gene were designated as *C. hominis* by *gp60* typing.

Using BLAST analysis, it was not possible to subtype the HCN1377 isolate which could only be identified by multiple alignment, and it was found that this isolate corresponded to the Id17 subtype (Table 3).

An overview of the positivity rates for *Cryptosporidium* spp., including its species and subtypes, stratified by age groups and selected risk factors, is provided. The variables presented were chosen based on their biological and clinical relevance, aiming to highlight key factors that may influence the distribution and potential health effects of the genotypes in the studied populations. These findings are summarized in Table 4.

**Figure 1:**
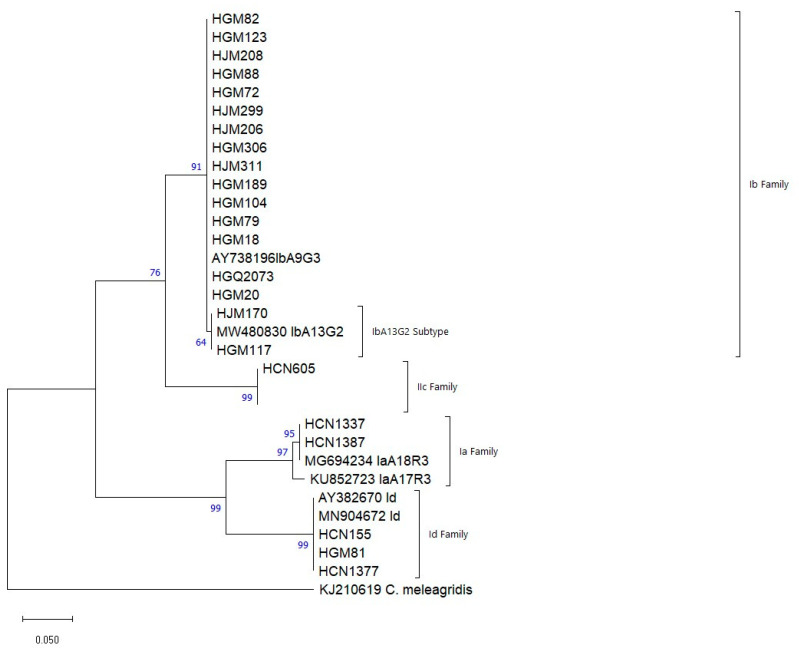
Phylogenetic relationships among different genotypes identified in children up to 14 years with *Cryptosporidium* spp. recruited in Maputo, Sofala, Zambézia and Nampula, during the years 2015–2019. The analysis was made using the Maximum Likelihood method, for the *gp60* gene (360 bp), between the positions 93 and 603 in relation to the reference sequences. Genetic distances were calculated using the 2-parameter Kimura model. *Cryptosporidium meleagridis* was used as an external group to root the tree.

The phylogenetic tree formed four clades. The first grouped the family Ib with its subtypes IbA9G3 and IbA13G2 and the reference AY738196. The second clade contains the single subtype IIcA5G3 of *C. parvum*, with reference MG694245. The third clade grouped the family Ia with subtypes IaA17R3 and IaA18R3 and its references MG694234.1 and KU852723.1, and the fourth grouped isolates of the family Id with references AY382670 and EF591785 (Figure 1).

### 3.4. Genotyping of G. duodenalis

The sequencing data of the *bg* gene was successfully obtained for 23 out of the 27 samples. The remaining four samples were excluded from subsequent analyses due to background noise in the chromatograms. A review of the forward and reverse chromatograms revealed small inconsistencies in signal intensity, suggesting potential issues with the sequencing reaction. However, the possibility of sample-related factors, such as low DNA quality or contamination, cannot be ruled out.

The analysis of the sequences allowed the identification of 65.2% (15/23) of isolates as assemblage A and 34.8% (8/23) as assemblage B. There was no identity among the sequences of the present study with those corresponding to the specific genotypes of animals: canine (C, D) ungulates (E) or feline specific (F).

### 3.5. Local BLAST Alignment and Sequence Identification

Among the 15 isolates of assemblage A, 13 (86.6%) showed 100% identity with the reference sequence AY072723 (AII) and 1 (6.6%) showed 100% identity with the reference AY072724 (AIII). One isolate (6.6%) presented an SNP in relation to the reference sequence AY072723, in position 345 (T/C).

Among the eight sequences grouped as being of assemblage B, seven (87.5%) belonged to subassemblage BIII and one (12.5%) to BIV. Of these, four were 100% identical to the reference sequence AY072727, and the remaining three showed differences of one SNP at positions 309 (T/C). The only BIV isolate found also showed a mutation at position 600 (T/C) when compared to reference sequence MT332785.1 of the Genbank (Table 5).

An overview of the positivity rates for *G. duodenalis*, including its subtypes and assemblages, stratified by age groups and selected risk factors, is provided. The variables presented were chosen based on their biological and clinical relevance, aiming to highlight key factors that may influence the distribution and potential health effects of the genotypes in the studied populations. These findings are summarized in Table 6.

The assemblages A and B were clearly positioned in two groups. Assemblage A grouped its subassemblages IIA, IIA and their respective gen bank references.

Assemblage B in turn grouped BIV, BIII and the Gen bank references.

**Figure 2:**
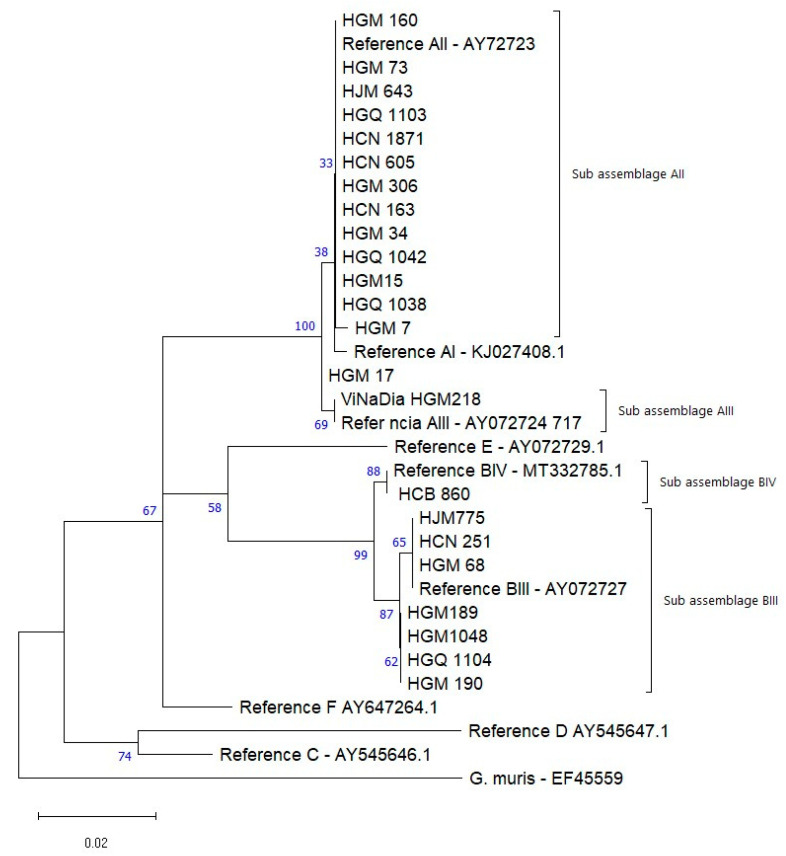
Phylogenetic relationships between the different genotypes identified in children up to 14 years with *G. duodenalis* recruited in Maputo, Sofala, Zambézia and Nampula, during the years 2015–2019. The analysis was performed by the Maximum Likelihood method for the *bg* gene (511 bp), between positions 93 and 603 in relation to the reference sequences. Genetic distances were calculated using the 2-parameter Kimura model. *Giardia muris* (access EF455599) was used as an external group to root the tree.

The isolates HGM17, HGM68, HJM775 and HCN251 present SNP in relation to the other isolates and references of the respective clusters (Figure 2).

## 4. Discussion

Based on parasitological data previously reported by the authors, 8.1% of the pediatric population was found to be infected with *Cryptosporidium* spp. [33]. This infection proportion was lower when compared to the 14.7% reported in the GEMS [4] but higher than the prevalence of 5.7% found in two public hospitals in Manhiça among children with malaria comorbidity [41]. Interestingly, no cases of *Cryptosporidium* spp. infection were detected in a study conducted in Magude in asymptomatic children [28]. Variations in prevalence were also noted in studies from Nampula and Zambézia, where *Cryptosporidium* spp. ranked as the third (3.4%) [42] and fifth (1.2%) [43] most commonly detected parasite in symptomatic children, respectively. Such differences in prevalence are not uncommon and can arise due to variability in geographic regions, study designs, sample sizes, age groups, HIV seroprevalence, sensitivity of the diagnostic methods used and local climatic conditions [22,44]. Furthermore, the opportunistic nature of *Cryptosporidium* should also be considered. Mozambique has some of the highest rates of HIV (12.6%) and malnutrition (38%) in Southern Africa, both of which compromise the immune system and increase susceptibility to infections [45]. Oocysts in tap water or contamination after collection likely contributed to the high infection rates reported [46]. As oocysts are resistant to chlorination, infections can persist despite water treatment, highlighting the need for alternative disinfection methods and further research.

Living in Beira, Sofala province, was a significant risk factor for *Cryptosporidium* infection, likely due to poverty, poor sanitation and frequent natural disasters such as floods and cyclones. These events disrupt water and health infrastructure, increasing the transmission of waterborne diseases [47,48]. Additionally, the association between illiterate mothers and *Cryptosporidium* infection in children suggests that lack of education may limit awareness of preventive practices. Similar findings were reported in Cameroon among children under 5 years [49]. The adjusted model showed that with each month of age, *Cryptosporidium* infection decreased by 2.8%. Children aged 12 to 23 months were the most prone to infection, highlighting *Cryptosporidium* as a significant cause of diarrhea in this age group. This increased susceptibility can be attributed to behavioral and hygiene-related factors, such as greater environmental exploration, frequent contact with contaminated surfaces and limited hygiene practices, particularly in settings with poor sanitation. Similar findings were reported in Egypt, where children under 12 months were 2.4 times more likely to be infected, and those aged 12–23 months were 1.9 times more likely to be infected compared to older children (*p* < 0.01) [50]. In Nigeria, children aged 1–2.5 years also had a higher prevalence, decreasing in older children [51]. A study in the Central African Republic showed infection rates of 16% in infants, 7% in young children and 9% in older children (*p*-value = 0.04) [52].

The prevalence of *G. duodenalis* (1.3%), reported previously by the authors in the population analyzed [33], is among the lowest reported in Mozambique for children with gastrointestinal symptoms. Higher rates were observed in studies using PCR, such as 52% in Zambézia [42] and 47% in Manhiça [27], as well as 72.2% in asymptomatic children in Magude [5]. Using ELISA, Nhampossa et al. [3] found rates of 10%, 23% and 35% in the 0–11, 12–24 and 24–59-month age groups, respectively. In contrast, studies using rapid diagnostic tests (RDTs) reported 29.3% prevalence in children from HIV and malnutrition wards at HCN [24], while microscopy detected lower rates, such as 6.7% in HCM [41]. The lower prevalence in our study could be attributed to the reduced sensitivity of microscopy, which depends on sample preservation and the microscopist’s expertise. At HCN, RDTs detected more than double the positivity compared to modified acid-fast staining (29.3% vs. 13.1%) [24], as RDTs detect antigens rather than intact forms [53]. The low prevalence observed may be attributed to the number of stool samples collected per participant and inconsistent adherence to CDC guidelines for specimen handling, such as timely preservation and refrigeration, which could have impacted trophozoite detection [54].

The higher prevalence of *G. duodenalis* in Maputo (18/19) positive samples may be partially attributed to logistical factors, as samples collected in this region were tested more promptly compared to other provinces. Living in traditional masonry houses was statistically associated (*p*-value = 0.002) with an increased risk of infection; however, all positive cases were reported in children living in this type of housing. This result should be cautiously interpreted, considering the unsanitary living conditions often observed in these households, such as poor waste management and limited hygiene practices, exacerbated by the region’s hot and humid climate [55]. Additionally, the absence of adequate water treatment (*p*-value = 0.04) may have facilitated the spread of *G. duodenalis,* suggesting water as a potential transmission route. In Zambezia, a study on intestinal parasites in school-age children found that drinking river/stream water, either as a primary or secondary source, was associated with *G. duodenalis* infection. However, this risk was mitigated by water treatment methods, such as chlorination or boiling [42].

In this study, molecular analysis revealed two *Cryptosporidium* species: *C. hominis* (77.8%) and *C. parvum* (18 isolates). The absence of amplification in other samples could be due to factors like low DNA quality, degradation during storage, or PCR inhibitors in the fecal samples, despite positive results by microscopy and/or ELISA. A study in Maputo found similar results, with *C. hominis* in 93% (27/29) and *C. parvum* in 3.5% (1/29) of cases [29]. Another study conducted in Manhiça in rural settings identified *C. hominis* in 79.6% (70/88) and *C. parvum* in 19.3% (17/88), along with *C. meleagridis* in 1.1% (1/88) of children under five years of age [6]. Similarly, *C. hominis* was the predominant species in a study conducted in Gaza (southern Mozambique), in HIV-positive patients [26]. These findings align with most African studies, where *C. hominis* is the predominant cause of infection. High frequencies of *C. hominis* were also reported in pediatric populations in Tanzania [56] and São Tomé and Príncipe [57].

In this study, three subtype families of *C. hominis* (Ia, Ib and Id) and one of *C. parvum* (IIc) were identified, with family Ib being the most common (62.5%). This suggests that anthroponotic, waterborne, or foodborne transmission is a major route of infection in children, emphasizing the importance of a One Health approach to integrated prevention and control strategies. Similar findings were reported in a *Cryptosporidium* subtyping study in rural Maputo (Manhiça), though a shift in the dominant genotype was observed between urban and rural areas [6]. The Ib and Id families were also found in Gaza in HIV-positive and/or tuberculosis patients [25]. However, the Ib family was not identified in a study at HCM in adult patients with diarrhea, though families Ia, IIc and IIe were documented [5]. The IbA9G3 and IbA10G2 subtypes are the most reported within the Ib family worldwide [58] and have previously been identified in Mozambique, suggesting that this family may be endemic. Infections with the Ib family are associated with nausea, vomiting, malaise, diarrhea and are commonly linked to waterborne outbreaks [59]. Despite efforts to improve water, sanitation and hygiene, the presence of the IbA9G3 subtype presents a significant challenge, as only 54% of the population has access to water. Many people rely on river basins and other bodies of water, which are often shared by animals, increasing the risk of oocyst spread, especially in rural and peripheral areas where agriculture and livestock farming are common [14,60,61]. Vomiting was the most common symptom in children infected with the Ib family (68.8%, 11/16) and was also observed in the only child co-infected with HIV and *Cryptosporidium* spp. (10%, 1/10). The IbA9G3 subtype, the most prevalent in our study, has also been observed in human cases in Kenya [62], Zambia [63] and Ethiopia [64], particularly in children with diarrhea. The Ia family subtypes have been reported in immunocompromised individuals with or without diarrhea in India [65], and in HIV-positive patients with gastrointestinal symptoms in Equatorial Guinea [66], but these subtypes have not been reported in Mozambique so far. Of the three Id family subtypes found in this study, two have been previously reported in the Manhiça district [6], while IdA17 is reported here for the first time in Mozambique. The Id family has been associated with diverse and severe clinical manifestations in immunocompromised patients in India [65] and Egypt in asymptomatic children [67]. The IIcA5G3a subtype of *C. parvum* appears to infect humans almost exclusively. A genomic evolutionary analysis of anthroponosis in *Cryptosporidium* showed that *C. parvum* split into two subclasses, with the anthroponotic subgenotype IIc-a clustering with a subtype of *C. hominis* and referred to as *C. p. anthroponosum,* contrasting with the zoonotic subtypes *C. p. parvum* [68]. In the present study, this isolate grouped with the Ib subtype with a bootstrap support of 76%, which could cause confusion as they belong to different species.

The prevalence of *G. duodenalis* assemblage A (AII) was 62.5%, similar to the 53% found in Magude. Previous studies in Mozambique using MLSG reported 90% assemblage B and 10% assemblage A [27], and 88.4% assemblage B [25]. In a study with HIV-positive patients, subassemblages AII and BIV were identified [26], while in the genotyping study at HCN, assemblage B predominated with 82.8% [24]. The differences observed could be due to the molecular approach and study population. Previous studies used MLSG with three loci, increasing detection sensitivity, unlike the present study, which analyzed only the bg locus. Additionally, studies in Zambézia and Angola showed a higher prevalence of assemblage B (asymptomatic children), with subassemblages BIII and BIV [69]. In this study, the subassemblage BIII was most common in Maputo, similar to the findings in Manhiça where BIII and BIV were frequent (13% and 14%, respectively) [27]. In Nampula, subassemblage AII was predominant (75%), whereas in a previous study by Ferreira, the AIII subtype was most reported (7%) [24]. In Sofala, the only isolate identified was BIV, which was previously reported in Zambézia, Gaza, and Maputo [25,26,27]. Subassemblage AII is mostly described in humans [70], suggesting that anthroponotic transmission is likely the main route of *Giardia* transmission in Mozambican children. However, zoonotic transmission, as well as environmental or contaminated food and water sources, cannot be ruled out, as both assemblages have been observed in animals [71,72]. Polymorphism in *G. duodenalis* assemblage B has been frequently reported in several studies, while assemblage A is considered rare in comparison [25,27,73]. In this study, one isolate (HGM17) showed mutations in different positions when compared to the AI, AII, and AIII references, and could not be grouped with any of these subassemblages. This might be due to sequencing failure or the presence of a new variant.

The key contribution of this study was the identification of new *C. hominis* subtypes in Mozambique, enhancing the understanding of the molecular epidemiology of *G. duodenalis* genotypes and *Cryptosporidium* previously described in the country. The molecular epidemiology of *G. duodenalis* in Sofala was previously unknown, and *Cryptosporidium* genotyping data were only available for Maputo. This study contributes new insights into *Cryptosporidium* genotyping in Zambézia and Nampula.

The study’s main limitations include the small number of isolates from Zambézia and Nampula and the absence of samples from Sofala due to logistical challenges. This restricted sample size hindered the analysis of regional differences in subtypes. Additionally, the lack of data on prior treatments likely led to an underestimation of *G. duodenalis* true frequency in the sampled children.

## 5. Conclusions

From the total of *Cryptosporidium spp.*-infected children, the higher infection rates were observed in children from Sofala and those with illiterate parents. *Cryptosporidium hominis* was the dominant species, with the IbA9G3 subtype suggesting anthropogenic, waterborne, or foodborne transmission routes. Additionally, 1.3% of children were infected with *G. duodenalis*, with higher risk among children from Maputo, particularly those whose families did not treat water and lived in masonry houses. The AII subassemblage of *G. duodenalis* was most prevalent in hospitalized children with diarrhea, indicating anthroponotic and/or waterborne or foodborne transmission.

These findings underscore the urgent need for integrated sanitation improvements and public health education to reduce transmission of these two protozoa as well as other pathogens found in this study. In a One Health context, this study emphasizes the importance of a collaborative, multisectoral approach to addressing sanitation, access to safe water and public health literacy. By joining efforts in the health, environment and community sectors, more effective action can be taken to prevent the spread of these pathogens, substantially improving health outcomes in vulnerable populations.

## Figures and Tables

**Table 1:** Sociodemographic features of 1424 children enrolled in the study.

*Cryptosporidium* spp.					*G. duodenalis*			
Variable	*n*/N	%	CI95%	*p*-Value	*n*/N	%	CI95%	*p*-Value
Gender				0.427 ^a^				0.972 ^a^
Male	63/830	7.6	(6.0; 9.6)		11/830	1.3	(0.7; 2.3)	
Female	52/594	8.8	(6.7; 11.3)		8/594	1.3	(0.7; 2.6)	
Age groups (months)				0.021 ^a^	7/682	1.0	(0.5; 2.1)	
0–11	59/682	8.7	(6.8; 11.0)		4/196	2.0	(0.8; 5.1)	
12–23	46/481	9.6	(7.3; 12.5)		0/65	0.0	(0; 00)	
24–59	10/196	5.1	(2.8; 9.1)		0	0.0	(0; 00)	
Province				0.038 ^a^				<0.001 ^b^
Maputo	71/745	9.5	(7.6; 11.9)		18/745	2.4	(1.5; 3.8)	
Sofala	8/78	10.3	(5.3; 19.0)		1/78	1.3	(0.2; 6.9)	
Zambézia	5/158	3.2	(1.4; 7.2)		0/158	0.0	(0; 00)	
Nampula	31/443	7.0	(5.0; 9.8)					
Source of water				0.195 ^b^				0.065 ^b^
Tap water at home	61/802	7.6	(6.0; 9.7)		16/802	2.0	(1.2; 3.2)	
Spring water	37/442	6.9	(6.1; 11.3)		1/442	0.2	(0.004; 1.3)	
Well water	10/144	6.9	(3.8; 12.3)		2/144	1.4	(0.38; 4.9)	
River/Lake/Lagoon	2/5	40.0	(11.8; 76.9)		0/5	0; 0	-	
Bottled water	0/4	(0; 00)	(0; 00)		0/4	0; 0	-	
Water Treatment				0.871 ^b^				0.040 ^b^
Boiling	23/243	9.5	(6.4; 13.8)		4/243	1.6	(0.6; 4.2)	
Chlorination	20/254	7.9	(5.2; 11.9)		1/254	0.4	(0.07; 2.2)	
Filtration	0/11	-	-		1/11	9.1	(1.6; 37.7	
Other	1/12	8.3	(1.5; 35.4)		1/12	8.3	(1.5; 35.4)	
Não	71/881	8.1	(6.4; 10.0)		12/881	1.4	(0.8; 2.4)	
Types of houses				0.058 ^b^				0.002 ^b^
Reed	8/55	14.5	(7.6; 26.6)		0/55	-	(0; 0)	
Mud	25/417	6.0	(4.1; 8.7)		0/417	-	(0; 0)	
Masonry	74/884	8.4	(6.7; 10.4)		19/884	2.15	(1.4; 3.3)	
Type of food				0.716 ^a^				0.918 ^b^
Breast milk	25/288	8.7	(6.0; 12.5)		5/288	1.7	(0.7; 4.0)	
Formula	7/100	7.0	(3.4; 13.6)		1/100	1.0	(0.2; 5.5)	
Mixed (breast milk and formula)	47/537	8.8	(6.7; 11.4)		6/537	1.1	(0.5; 2.4)	
Other	33/471	7.0	(5.0; 9.7)		6/471	1.3	(0.6; 2.8)	
Education level of mother				0.036 ^a^				0.895 ^b^
Illiterate	22/168	13.1	(8.8; 19.0)		2/168	1.2	(0.3; 4.2)	
Primary	39/509	7.7	(5.7; 10.3)		8/509	1.6	(0.8; 3.1)	
Secondary/above	52/726	7.2	(5.5; 9.3)		9/726	1.2	(0.7; 2.3)	
Mother’s marital status				0.058 ^b^				0.095 ^b^
Married	63/875	7.2	(5.7; 9.1)		8/875	0.9	(0.5; 1.8)	
Single	48/470	10.2	(7.8; 13.3)		11/470	2.3	(1.3; 4.1)	
Divorce d/widow	1/45	2.2	(0.4; 11.6)		0/45	0.0	(0; 00)	
Child caregiver				0.265 ^b^				0.591 ^b^
Mother	104/1257	8.3	(6.9; 9, 9)		16/1257	1.3	(0.8; 2.1)	
Father	1/27	3.7	(0.7; 18, 3)		0/27	0; 0	(0; 00)	
Uncle/aunt	3/16	18.8	(6.6; 43, 0)		0/16	0; 0	(0; 00)	
Grandparents	5/58	8.6	(3.7; 18, 6)		1/58	1.7	(0.3; 9.1)	
Brothers	0/5	(0; 00)	(0; 00)		0/5	0.0	(0; 00)	
Babysitter	0/32	(0; 00)	(0; 00)					
Agriculture practice				0.363 ^a^				1.000 ^b^
Yes	11/169	6.5	(3.7; 11.3)		2/169	1.2	(0.3; 4.2)	
No	96/1119	8.6	(7.1; 10.4)		13/1119	1.2	(0.7; 2.0)	

^a^ chi-square test, ^b^ Fisher’s exact test.

**Table 2:** Risk factors associated with cryptosporidiosis in the study.

Variable	*n*/N	%	CI95%	*p*-Value
	*p*-value	OR (IC 95%)		0.427 ^a^
Age	0.006	0.973 (0.955–0.992)	(6.0; 9.6)	
Province				
Maputo	0.028	2.840 (1.121–7.199)	(6.7; 11.3)	0.021 ^a^
Sofala	0.035	3.499 (1.095–11.185)	(6.8; 11.0)	
Zambézia	0.154	2.021 (0.768–5.322	(7.3; 12.5)	
Nampula	0.070	Ref.	(2.8; 9.1)	
Education level of mother				0.038 ^a^
Illiteracy	0.005	2.150 (1.252–3.690)	(7.6; 11.9)	
Primary	0.628	1.113 (0.721–1.720)	(5.3; 19.0)	
Secondary/above	0.018	Ref.	(1.4; 7.2)	

^a^ chi-square test.

**Table 3:** Genetic diversity at the *gp60* gene of *Cryptosporidium* spp. identified in the study.

Gene	Isolate	Family	Subtype	Reference	Extension
Gp60	HGM18, HGM20, HGM72, HGM79, HGM82, HGM88, HGM104, HGM123, HGM189, HJM206, HJM208, HJM299, HGM306, HJM311, HGQ2073	Ib	IbA9G3	AY738196	15–399
	HGM117, HJM170	Ib	IbA13G2	MW480830	18–414
	HCN1337	Ia	IaA18G2R2	MG694234.1	12–360
	HCN1387	Ia	IaA17G2R2	KU852723.1	24–363
	HGM81	Id	IdA20	JX088404.1	24–473
	HCN155	Id	IdA21	MN904672.1	15–477
	* HCN1377	Id	IdA17	AY382670	12–478
	HCN605	IIc	IIcA5G3a	MN904722	19–349

* Isolate with discrepancies between the two alignment methods. The extension represents the comparison sites between the study sequences with the GenBank reference sequences.

**Table 4:** Positivity rates of *Cryptosporidium* spp., including its species and subtypes stratified by age groups and selected risk factors.

.	*Cryptosporidium* spp.	*C. hominis*			*C. parvum*
Variable	Total	Ia	Ib	Id	IIc
Gender	*n* = 23 (%)	*n* = 2 (%)	*n* = 17 (%)	*n* = 3 (%)	*n* = 1 (%)
Male	14 (60.9)	0 (0.0)	11 (64.7)	2 (66.7)	1 (100)
Female	9 (39.1)	2 (100)	6 (35.3)	1 (33.3)	0 (0.0)
Age groups (months)	*n* = 23 (%)	*n* = 2 (%)	*n* = 17 (%)	*n* = 3 (%)	*n* = 1 (%)
0–11	16 (69.4)	2 (100)	11 (64.7)	2 (66.7)	1 (100)
12–23	5 (21.7)	0 (0.0)	5 (29.4)	0 (0.0)	0 (0.0)
24–59	1 (4.3)	0 (0.0)	1 (5.9)	0 (0.0)	0 (0.0)
60–168	1 (4.3)	0 (0.0)	0 (0.0)	1 (33.3)	0 (0.0)
Province	*n* = 23 (%)	*n* = 2 (%)	*n* = 17 (%)	*n* = 3 (%)	*n* = 1 (%)
Maputo	17 (73.9)	0 (0.0)	16 (94.1)	1 (33.3)	0 (0.0)
Zambézia	1 (4.3)	0 (100)	1 (5.9)	0 (0.0)	0 (0.0)
Nampula	5 (21.7)	2 (100)	0 (0.0)	2 (66.7)	1 (100)
Animal contact	*n* = 23 (%)	*n* = 2 (%)	*n* = 17 (%)	*n* = 3 (%)	*n* = 1 (%)
Yes	8 (34.8)	2 (100)	5 (29.4)	1 (33.3)	0 (0.0)
No	15 (65.2)	0 (0.0)	12 (70.6)	2 (66.7)	1 (100)
Diarrhea in the last 7 days	*n* = 14 (%)	*n* = 2 (%)	*n* = 9 (%)	*n* = 2 (%)	*n* = 1 (%)
Yes	2 (14.3)	1 (50.0)	0 (0–0)	0 (0.0)	1 (100)
No	12 (85.7)	1 (50.0)	9 (100)	2 (100)	0 (0.0)
Vomiting	*n* = 22 (%)	*n* = 2 (%)	*n* = 16 (%)	*n* = 3 (%)	*n* = 1 (%)
Yes	14 (63.3)	1 (50.0)	11 (68.8)	2 (66.7)	0 (0.0)
No	8 (36.4)	1 (50.0)	5 (31.3)	1 (33.3)	1 (100)
Fever	*n* = 22 (%)	*n* = 2 (%)	*n* = 16 (%)	*n* = 3 (%)	*n* = 1 (%)
Yes	11 (50.0)	1 (50.0)	8 (50.0)	1 (33.3)	1 (100)
No	11 (50.0)	1 (50.0)	8 (50.0)	2 (66.7)	0 (0.00)
HIV	*n* = 14 (%)	*n* = 2 (%)	*n* = 10 (%)	*n* = 1 (%)	*n* = 1 (%)
Yes	1 (7.1)	0 (0.0)	1 (10.0)	0 (0.0)	0 (0.0)
No	13 (92.9)	2 (100)	9 (90.0)	1 (100)	1 (100)

**Table 5:** Genetic variability of the *β-Giardin* gene of *G. duodenalis* isolates identified in the study.

Gene	Isolate	Family	Assemblage	Reference	Extension	ID of the Sequence
*β-giardin*	HGM160, HGM73, HJM643, HGQ1103, HCN1871, HCN605, HGM306, HCB163, HGM34, HGQ1042, HGM15, HGQ1038, HGM7.	A	AII	AY072723	93–603	MG736240.1
	HGM218	A	AIII	AY072724	93–603	FJ472824.1
	HGM17	A	Unknown		93–603	-
	HJM775, HCN251 HGM68. * HGM189 HGM1048, HGQ1104 e HGM190	B	BIII	AY072727	93–603	LC508615.1
	HGB860	B	BIV	MT332785.1	93–603	MK033096.1

* Isolates with mutation at position 309 (T/C). The extension represents the sites of comparison between the study sequences in relation to the reference sequences. The sequence ID represents the Genbank sequence reference.

**Table 6:** Positivity rates of *G. duodenalis* subtypes and assemblages stratified by age groups and selected risk factors.

	*G. duodenalis*	Assemblage A		Assemblage B		
Variables	Total	AII	AIII	BIII	BIV	Not Identified
Gender	*n* = 23 (%)	*n* = 13 (%)	*n* = 1 (%)	*n* = 7 (%)	*n* = 1 (%)	*n* = 1 (%)
Male	11 (47.8)	7 (53.8)	1 (100)	2 (28.6)	0 (0.0)	1 (100)
Female	12 (52.2)	6 (46.2)	0 (0.0)	5 (71.4)	1 (100)	0 (0.0)
Age groups (months)	*n* = 23 (%)	*n* = 13 (%)	*n* = 1 (%)	*n* = 7 (%)	*n* = 1 (%)	*n* = 1 (%)
0–11	9 (39.1)	6 (46.2)	1 (100)	1 (14.3)	1 (100)	0 (0.0)
12–23	9 (39.1)	3 (23.1)	0 (0.0)	5 (71.4)	0 (0.0)	1 (100)
24–59	4 (17.4)	3 (23.1)	0 (0.0)	1 (14.3)	0 (0.0)	0 (0.0)
60–159	1 (4.3)	1 (7.7)	0 (0.0)	0 (0.0)	0 (0.0)	0 (0.0)
Province	*n* = 23 (%)	*n* = 13 (%)	*n* = 1 (%)	*n* = 7 (%)	*n* = 1 (%)	*n* = 1 (%)
Maputo	14 (60.9)	7 (53.8)	1 (100)	5 (71.4)	0 (0.0)	1 (100)
Sofala	1 (4.3)	0 (0.0)	0 (0.0)	0 (0.0)	1 (100)	0 (0.0)
Zambézia	4 (17.4)	3 (23.1)	0 (0.0)	1 (14.3)	0 (0.0)	0 (0.0)
Nampula	4 (17.4)	3 (23.1)	0 (0.0)	1 (14.3)	0 (0.0)	0 (0.0)
Animal contact	*n* = 23 (%)	*n* = 13 (%)	*n* = 1 (%)	*n* = 7 (%)	*n* = 1 (%)	*n* = 1 (%)
Yes	9 (39.1)	6 (46.2)	0 (0.0)	3 (42.9)	0 (0.0)	0 (0.0)
No	14 (60.9)	7 (53.8)	1 (100)	4 (57.1)	1 (100)	1 (100)
Diarrhea in the past 7 days	*n* = 16 (%)	*n* = 11 (%)	*n* = 1 (%)	*n* = 4 (%)	*n* = 0 (%)	*n* = 0 (%)
Yes	2 (12.5)	2 (18.2)	0 (0.0)	0 (0.0)	0 (0.0)	0 (0.0)
No	14 (87.5)	9 (81.8)	1 (7.1)	4 (100)	0 (0.0)	0 (0.0)
Vomiting	*n* = 21 (%)	*n* = 12 (%)	*n* = 1 (%)	*n* = 8 (%)	*n* = 1 (%)	*n* = 1 (%)
Yes	12 (57.1)	7 (58.3)	0 (0.0)	3 (50.0)	1 (100)	1 (100)
No	9 (42.9)	5 (41.7)	1 (100)	3 (50.0)	0 (0.0)	0 (0.0)
Fever	*n* = 22 (%)	*n* = 12 (%)	*n* = 1 (%)	*n* = 7 (%)	*n* = 1 (%)	*n* = 1 (%)
Yes	9 (40.9)	6 (50.0)	0 (0.0)	2 (28.6)	0 (0.0)	1 (100)
No	13 (59.1)	6 (50.0)	1 (100)	5 (71.4)	1 (100)	0 (0.0)
HIV	*n* = 17 (%)	*n* = 10 (%)	*n* = 1 (%)	*n* = 4 (%)	*n* = 1 (%)	*n* = 1 (%)
Yes	1 (5.9)	1 (10.0)	0 (0.0)	0 (0.0)	0 (0.0)	0 (0.0)
No	16 (94.1)	9 (90.0)	1 (100)	4 (100)	1 (100)	1 (100)

Number of positives in the category (*n*) and (%) frequency.

## Data Availability

The original contributions presented in this study are included in the article. Further inquiries can be directed to the corresponding author.
